# Regulation of Hemichannels and Gap Junction Channels by Cytokines in Antigen-Presenting Cells

**DOI:** 10.1155/2014/742734

**Published:** 2014-09-09

**Authors:** Pablo J. Sáez, Kenji F. Shoji, Adam Aguirre, Juan C. Sáez

**Affiliations:** ^1^Departamento de Fisiología, Pontificia Universidad Católica de Chile, Alameda 340, 6513677 Santiago, Chile; ^2^Instituto Milenio, Centro Interdisciplinario de Neurociencias de Valparaíso, Pasaje Harrington 287, Playa Ancha, 2360103 Valparaíso, Chile

## Abstract

Autocrine and paracrine signals coordinate responses of several cell types of the immune system that provide efficient protection against different challenges. Antigen-presenting cells (APCs) coordinate activation of this system via homocellular and heterocellular interactions. Cytokines constitute chemical intercellular signals among immune cells and might promote pro- or anti-inflammatory effects. During the last two decades, two membrane pathways for intercellular communication have been demonstrated in cells of the immune system. They are called hemichannels (HCs) and gap junction channels (GJCs) and provide new insights into the mechanisms of the orchestrated response of immune cells. GJCs and HCs are permeable to ions and small molecules, including signaling molecules. The direct intercellular transfer between contacting cells can be mediated by GJCs, whereas the release to or uptake from the extracellular milieu can be mediated by HCs. GJCs and HCs can be constituted by two protein families: connexins (Cxs) or pannexins (Panxs), which are present in almost all APCs, being Cx43 and Panx1 the most ubiquitous members of each protein family. In this review, we focus on the effects of different cytokines on the intercellular communication mediated by HCs and GJCs in APCs and their impact on purinergic signaling.

## 1. Introduction

An efficient immune response against pathogens and other challenges requires efficient coordination between different cell types, making cell-cell interaction a key step [1, 2]. To this end, the immune system uses different types of cellular communication, being the autocrine and paracrine signaling mediated by cytokines two of the most studied ones [3]. These types of signaling allow communication not only among immune cells, but also with resident cells of challenged tissues [4]. This coordination plays a pivotal role in antigen-presenting cells (APCs) activation because they specifically trigger activation of other cells through immunological synapse, such as T- and B-cell activation that mediate adaptive immunity [5], and the cytokines released at this stage determine the onset of the immune response [6].

Cytokines are soluble or membrane-attached proteins that have pro- or anti-inflammatory properties and are produced by immune and nonimmune cells. As expected, the abnormal release of cytokines promotes the development and progression of pathological conditions with rather diverse etiologies, including rheumatoid arthritis, cancer, and even depression [7–9]. In addition, cytokines favor other types of cellular communication through the expression of cell surface molecules [10] and/or release of soluble molecules, as we discuss in the next section. Both of these alternative mechanisms of cellular communication, which are dependent or independent of cellular contacts, might occur through membrane channels constituted by connexins (Cxs) or pannexins (Panxs).

Nowadays, immunologists' rising interest in Cx- and Panx-based channels is evident in the literature. One of the relevant findings that put GJCs in the center of the immunology field is the contribution to inflammation, antigen presentation, tolerance, HIV sensing, and tumoral immunity [11–17]. Here, we review the cytokine regulation of GJCs and HCs in different APCs.

### 1.1. Gap Junction Channels and Hemichannels

**Figure 1:**
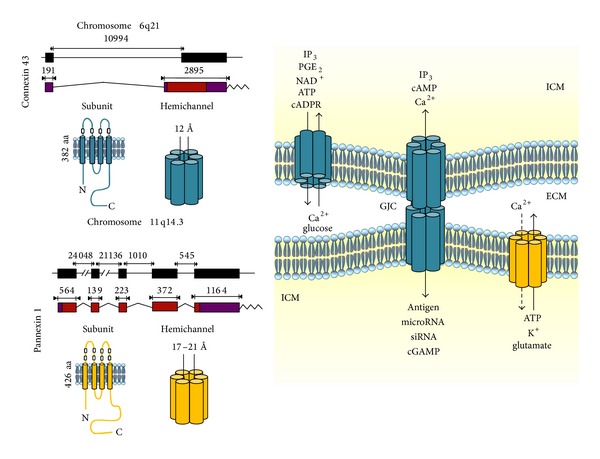
Connexin 43 and pannexin1 at gene and protein levels. Left: a diagram depicting the genomic regions, mRNA, and membrane topology of human connexin 43 (Cx43, top left) and pannexin 1 (Panx1, bottom left). Genomic loci are represented by black boxes that stand for the corresponding exons. mRNA diagrams representing the exons as coding protein regions (red boxes) and 3′- and 5′-non-coding areas (purple boxes) are shown. The intron lengths are indicated in the schemes of genomic loci, and exon sizes are indicated in the mRNA diagrams. In the membrane topology the white squares indicate extracellular cysteine residues of each protein. Six protein subunits constitute a hemichannel (HC), which has different pore sizes. Right: two adjoining cells forming a gap junction channel (GJC) at the cell interface. Each cell presents HCs formed by Cx43 or Panx1. Arrows denote the bidirectional communication with the intracellular milieu (ICM) for GJCs and the extracellular milieu (ECM) for HCs; some immunorelevant molecules are shown. Dotted line for Ca^2+^ permeating Panx1 HCs indicates that this phenomenon is not fully demonstrated.

The most studied mechanism of intercellular communication that depends on close cell-cell contact is mediated by gap junction channels (GJCs) [18]. Since most immune cells are generally sparse within tissues, it is possible that this feature delayed the studies on GJCs. Members of the Cx family share the membrane topology and number of units that oligomerize in a GJC (dodecamer) and show high homology in primary sequence (Figure 1) [18–20]. These GJCs are formed by the docking of two adjacent hemichannels (HCs, hexamers) and allow direct contact-dependent cellular communication because they are permeable to ions and small compounds including immunorelevant molecules [13, 21–26].

The turnover of Cxs is between 2 and 3 h indicating that the strength of intercellular communication can be quickly affected by changes in rate of synthesis and/or degradation of GJC protein subunits. In addition, closure of GJCs can be induced in a few seconds by changes in the state of phosphorylation of Cxs [18]. Therefore, the high plasticity of GJCs is compatible with transient as well as stable gap junctional communication between contacting cells.

Recently, another family of proteins named Panxs and constituted by only three members (Panx1–3) was proposed to form GJCs. Exogenous expression of Panx1 alone or with Panx2 establish GJCs in oocytes [27]. Similar findings were obtained with Panx1 expression in mammalian cells [28]. Moreover, Panx3 has been proposed to form GJCs between osteoblasts and to contribute to the differentiation of C_2_C_12_ cells into osteoblasts [29]. However, the expression of functional Panx GJCs still remains controversial [30]. Panxs share their membrane topology but show only very little homology in their primary sequence (Figure 1). In addition, Cx and Panx HCs are oligohexamers [18], but Panx2 has been shown to form octamers [31].

HCs are the least studied autocrine/paracrine intercellular communication pathway mainly due to their rather recent discovery. They correspond to one-half of a GJC, and Cx and/or Panx HCs are present in the cell surface of all cells so far studied, allowing the exchange of ions and small molecules between the intra- and extracellular compartments [20]. Cx and Panx HCs differ in their regulation and pore size [18, 20, 31]. Panx1 HCs exhibit a bigger pore vestibule, but the pore neck seems to be more selective than that of Cx HCs since they are not permeable to anionic molecules >250 Daltons [32], whereas several Cx HCs are permeable to Evans blue (−4 negative charge and ~950 Daltons) [33]. HCs allow communication in a cell-cell contact-independent manner because they permit the release or uptake of small molecules [34, 35].

Several conditions increase the open probability of Cx HCs including reduction of extracellular or increase of intracellular Ca^2+^ concentration [36]. In contrast, Panx1 HCs are not directly affected by changes in extracellular Ca^2+^ concentration, but extracellular ATP activation of some P2Y or P2X_7_ receptors induces opening of Panx1 HCs [37]. Several GJC blockers also inhibit Cx and/or Panx HCs. Extracellular La^3+^ does not block Cx GJCs [38] or Panx HCs [39] but blocks all Cx HCs so far studied. Nevertheless the use of La^3+^ should be accompanied by using other blockers because it has been shown to block other membrane channels [40].

Cx43 and Panx1, the most ubiquitous members of each family of HC forming proteins, are expressed in APC [14, 20, 34, 41, 42]. Cytokine regulation of intercellular communication through GJCs and HCs might contribute to a rapid amplification and coordination of activating or inhibitory signals among neighboring cells. Here, we summarize the current knowledge on the regulation of both types of cellular communication by cytokines.

### 1.2. Immunorelevant Molecules and Cx- and Panx-Mediated Cell-Cell Communication

The study of GJCs began in the early 60s with the description of the structure responsible for intercellular electrical transmission [43]. These studies showed current transference between contacting excitable cells and were the first to use the term gap junction to identify this structure [44–47]. In the 70s, the permeation of different immunorelevant molecules was described. These studies included small peptides [48], IP_3_ [49], and cAMP [50], but the study of Cxs and Panxs in immune cells had to wait for almost 30 years to be reported.

Although the presence of GJCs at ultrastuctural level was shown at the end of the 80s during antigen presentation [38, 51, 52], immunologists put an eye on the GJC field after the demonstration of antigen transfer (linear peptides up to 1,800 kDa) through GJCs in APCs [25]. This direct antigen transfer through GJCs allows cross-presentation, which corresponds to presentation of antigens in major histocompatibility complex (MHC) class I molecules by APCs that acquire antigens from infected or tumoral cells and after presentation to T cells initiating an effective immune response [24, 25]. Following this, our group and collaborators were able to show the transference of tumoral antigens between dendritic cells after stimulation with tumoral necrosis factor-α (TNF-α) and tumoral lysate [24]. In addition, GJCs allow cell-cell transference of single- and double-stranded RNA [53], as well as specific single-stranded microRNA [23], which has a high impact on immune responses [54]. Recently, the cell-cell transference of two different microRNAs (miR-142 and -223) between macrophages was demonstrated [55]. These data open new unexplored fields in the study of GJCs, which might be used for specific delivery of microRNA and siRNA. Whether Cx or Panx HCs allow the transfer of single- or double-stranded RNA has to be studied.

Ca^2+^ signaling plays a pivotal role in immune cells and contributes to all stages of the immune response. In APCs, it contributes directly to their migration, maturation, and cell death [56]. The transference of second messengers associated with Ca^2+^ signaling, such as IP_3_, through GJCs was demonstrated several years ago [22, 49]. In addition, IP_3_ is released through HCs [57], and intracellular IP_3_ contributes to increase HC activity [58]. IP_3_ contributes to different steps of the immune response [59] and plays an important role in migration of dendritic cells (DCs) [60]. Then, it is possible to hypothesize that transference of IP_3_ between communicating DCs or release/uptake through HCs might have an impact on the phenotype of DCs. Moreover, direct Ca^2+^ transfer occurs via GJCs [26], and it is possible that similar Ca^2+^ communication occurs through DC-T-cell GJCs during immune synapse [13, 61]. In addition, it was shown recently that Cx [36, 62–67] and Panx HCs [28, 29] provide a new route for Ca^2+^ entry into the cell. Then, the functional expression of HCs in immune cells might also contribute to the Ca^2+^ signaling.

CD38 is an ectoenzyme expressed by myeloid and lymphoid cells that use NAD^+^ to generate cADPR and ADP-ribose, which contribute to several immune cell responses [68]. Interestingly, NAD^+^ permeates GJCs [22, 69] and Cx HCs [22, 70], and upon activation of P2X_7_ receptors increase the opening of Panx1 HCs [71]. It can be anticipated that NAD^+^ transfer or release through these channels might contribute to cell-cell communication in different immune cells. In addition, cADPR uptake occurs through Cx43 HCs [72], which in turn contributes to microglial survival [73].

ATP is a recognized DAMP that activates immune cells and also contributes to autocrine and paracrine activation when released from cells [34, 74, 75]. The contribution of Cx and Panx HCs to purinergic signaling has been reported and was recently revised [76]. Thus, Cx and Panx HC-mediated ATP release might play a role in all steps of the immune response. In contrast to ATP, prostaglandins (PGs) are small soluble molecules that seem to contribute to anti-inflammation in APCs, although this feature depends on the microenvironmental signals [77]. In particular, PGE_2_ contributes to induce gap junctional communication [78] and also is released through Cx43 HCs [79]. Moreover, PGE_2_ and purinergic signaling contribute to interleukin (IL) 1*β* release from macrophages [80]. Thus, PGE_2_ and other metabolites produced by cyclooxygenase-2 might be released from APCs (and/or other immune cells), which produce a different signature in the involved cells depending on the inflammatory mediators that coexist with them.

Description of functional GJCs in T cells occurred almost 4 decades ago [81, 82]. However, a rising interest in T-cell GJCs began very recently after the discovery of their role in regulatory T cells- (Tregs-) mediated tolerance [12]. GJCs allow cAMP transfer from Tregs to naïve T cells and provide immunosuppression [12]. In addition, GJCs between DCs and Tregs contribute to prevent the activation of CD8^+^ T cells [15], showing that GJCs provide amplification of activating or inhibitory signals.

**Figure 2:**
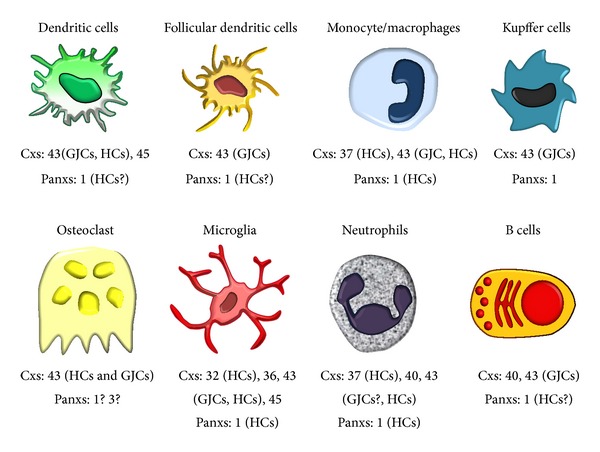
Connexin and pannexin expression in antigen-presenting cells. Summary scheme shows the expression of gap junction channels (GJCs) and hemichannels (HCs) formed by connexins (Cxs) and pannexins (Panxs) in different antigen-presenting cells (APCs). Question marks next to a protein (Cx or Panx) or channel type (GJC or HC) indicate that the expression or function remains unknown or is not fully shown.

The role of Cx- and Panx-based channels in infectious diseases is well documented [83], but an unexpected role was recently shown in the development of HIV infection. Cytosolic DNA-sensing occurs through an enzyme called cyclic guanosine monophosphate-adenosine monophosphate (cGAMP) synthase (cGAS) [84], which produces the second messenger cGAMP that enables DCs to sense HIV [85]. Importantly, transfer of cGAMP occurs through GJCs between Cx43 and Cx45 overexpressing cells [21]. This spreading of cGAMP activates STING (from stimulator of IFN genes) in the receiving cell, which in turn produces interferon (IFN) [21]. Since DCs and other APCs express Cx43 and Cx45 (Figure 2) [24, 86, 87], it is possible that gap junctional communication between these cells contributes to the HIV immune response.

## 2. Expression of Cxs in Antigen-Presenting Cells

Although GJCs in immune cells were described in the early 70s by Hülser and Peters who reported gap junctional communication between T cells [81, 82], the study of Cxs in APCs had to wait until the end of the decade when expression of GJCs and gap junctional communication was shown in macrophages [88, 89]. Later, they were found in DCs [51, 52, 90] and follicular DCs [91–93]. On the other hand, the study of HCs in the immune system started several years ago. Later in the 90s, Alves et al. (1996) showed ATP-induced dye uptake in macrophages, which was suggested to be mediated by HCs [94]. This study was followed by studies in microglia, neutrophils, and T cells several years later [34, 41, 95, 96].

### 2.1. Dendritic Cells (DCs)

**Figure 3:**
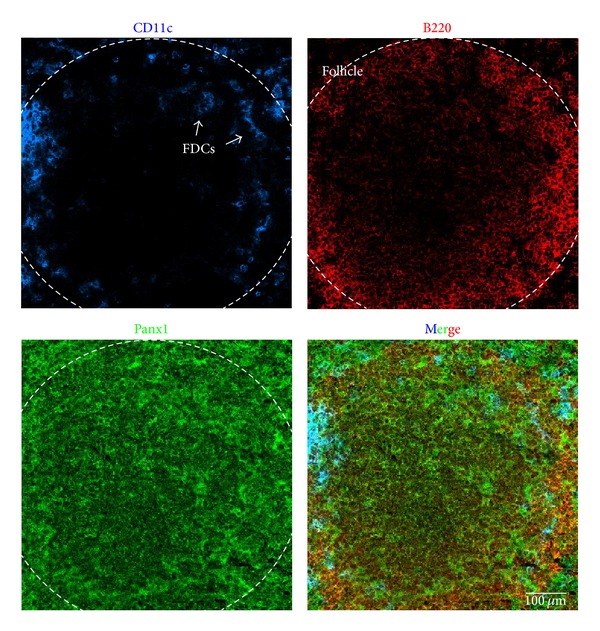
Dendritic and B cells of mouse spleen present pannexin1. Immunofluorescence of adult mice spleen cryosections (8 *μ*m thick) fixed in ethanol (70% v/v) at −20°C for 20 min, mounted in Fluoromount-G and observed in a confocal microscope (Olympus, FluoView FV1000). Pannexin1 (Panx1 in green: primary antibody: rabbit anti-Panx1 antibody and secondary antibody goat anti-rabbit IgG conjugated to FITC) immunoreactivity is shown. Cells were identified by their reactivity to specific antigens as follows: dendritic cells (DCs) by CD11c (blue, monoclonal mouse antibody conjugated to allophycocyanin) and B cells by B220 (red, monoclonal mouse antibody conjugated to phycoerythrin) in a follicle. Arrows denote follicular DCs (arrows). Merge is also shown. Scale bar: 100 *μ*m.

Ralph Steinman in the early 70s discovered the DCs [97], which emerge in the bone marrow from a myeloid common precursor and populate different organs [98]. In these cells, the expression of Cxs has been demonstrated, but the expression of Panxs remains unknown. However, the expression of Panx1 might be predicted by the ATP-induced dye uptake observed in these cells [99–102]. In addition, Panx1 expression has been detected at the mRNA level in DCs under resting conditions, while its upregulation has been demonstrated upon exposure to bacterial lipopolysaccharide (LPS) or IFN-*γ* in DCs [103, 104]. LPS-induced IL-1*β* release in DCs occurs in a P2X_7_ receptor-independent way [105], suggesting that P2X_7_ receptor-mediated opening of Panx1 might not contribute to inflammasome activation. However, whether Panx1 might contribute to other responses in DCs has not been reported yet. Here, we present evidence of Panx1 presence in CD11c^+^ DCs from mouse spleen (Figure 3).

In murine and human DCs (primary cultures and cell lines), the expression of Cx43 and Cx45 has been demonstrated at the mRNA and protein levels (Figure 2) [13, 15, 24, 61, 86, 87, 93, 106, 107]. In addition, migratory DEC205^+^ DCs, found at draining lymph nodes after muscle damage, show increased immunoreactivity for Cx43 and Cx45 [86]. Consistent with the requirement of cell activation for Cx expression, Cx43 was not detected in skin DCs under resting conditions [108]. Similarly, Cx43 was found to contribute to the establishment of oral tolerance, because it mediates antigen transfer from CD103^+^ DCs to macrophages in murine intestine [106]. Accordingly, expression of Cxs and functional state of GJCs are modulated by different cytokines (Table 1).

TNF-α is a proinflammatory cytokine and possibly the most relevant one because it is the first cytokine released by different cell types, including DCs, after exposure to different stimuli, such as cell damage or infection, and its receptor is expressed by all APCs [109]. However, TNF-α alone does not increase Cx43 total protein levels in murine or human DCs [24, 86] but potentiates the expression of functional GJCs between cultured DCs in combination with IL-1*β* or a tumoral lysate (Table 1) [24, 86]. Whether TNF-α induces HC activity in DCs remains unknown.

IL-1*β*, another proinflammatory cytokine released by different cell types including APCs, is maintained as an inactive precursor and after cleavage is released as a mature bioactive form to the extracellular milieu [110]. Similar to TNF-α, IL-1*β* alone does not induce gap junctional communication or Cxs expression but, in combination with TNF-α, induces GJCs and increases Cx43 and Cx45 levels in DCs (Table 1) [86]. The possible effect of IL-1*β* on the expression of HCs in DCs has not been reported yet.

**Figure 4:**
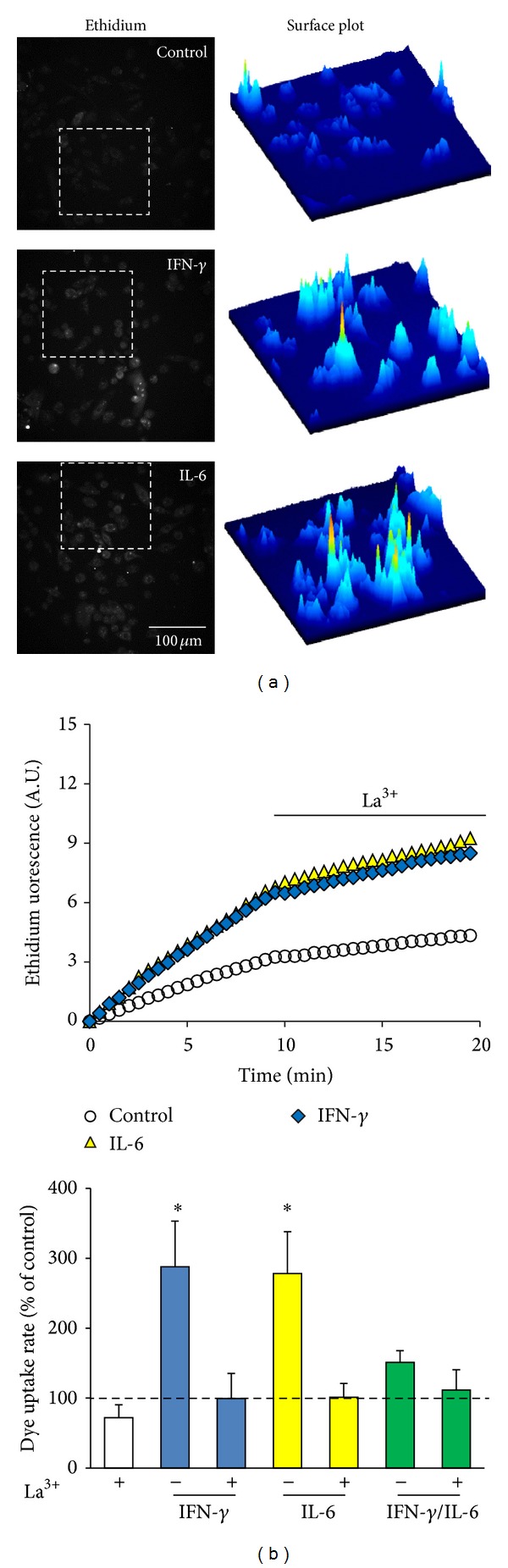
IFN-*γ* or IL-6 increases dye uptake in dendritic cells. Bone-marrow-derived dendritic cells (BMDCs) from balb/c mice were differentiated with 40 ng/mL GM-CSF and IL-4 for 7 days. At day 7, BMDCs were treated for 6 h with IFN-*γ* (10 ng/mL), IL-6 (10 ng/mL), or both, and ethidium uptake was evaluated in time-lapse experiments (Olympus BX 51W1I). (a) Left: fluorescence images of ethidium after 9 min of uptake. Scale bar: 50 *μ*m. Right: ImageJ surface plot analysis of fluorescence intensity of the region indicated in the field (dotted square). (b) Top: time-lapse ethidium uptake under control conditions (white circles) or after 6 h treatment with IL-6 (yellow triangles) or IFN-*γ* (blue diamonds). Each point corresponds to the mean of 30 cells. After 10 min of recording, La^3+^ (200 *μ*M) was added to the bath solution to block connexin hemichannels. Bottom: graph showing the basal dye uptake rate and the effect of La^3+^ on BMDCs after treatment with IFN-*γ* (blue bars), IL-6 (yellow bars), or both (green bars). Each bar corresponds to the mean ± SE (% of control condition, dotted line) of 3 independent experiments.

IFN-*γ* contributes to the control of viral infections and is mostly produced by T and natural killer (NK) cells, but it is also produced and released by DCs [111–114]. Similar to TNF-α and IL-1*β*, treatment with IFN-*γ* does not induce gap junctional communication or increase in Cx43 levels [87] but, in combination with TNF-α and IL-1*β*, promotes a synergic response on Cx43 and Cx45 levels in DCs [86]. Moreover, in combination with LPS, IFN-*γ* potentiates the functional expression of GJCs in DCs [87] and prolongs the TNF-α/IL-1*β*-induced dye coupling [86], showing that IFN-*γ* is an enhancer rather than inducer of gap junctional communication. In addition, we show here that IFN-*γ* induces dye uptake sensitive to La^3+^, suggesting that the IFN-*γ*-induced dye uptake is mediated by Cx HCs (Figures 4(a) and 4(b)).

IL-6, described initially as a stimulating factor for IgG production in B cells, is a cytokine produced by almost all nucleated cells [115] and drives T helper 17 (Th17) differentiation and inhibits Tregs [116, 117]. However, IL-6 also shows anti-inflammatory effects as it decreases the reducing immune response and promotes the release of anti-inflammatory cytokines after exercise, such as IL-10 and transforming growth factor-*β* (TGF-*β*) [118, 119]. From the GJCs perspective, IL-6 has an anti-inflammatory effect because it prevents the TNF-α/IL-*β*- and TNF-α/IL-*β*/IFN-*γ*-induced gap junctional communication in DCs [86]. Similar findings related to cytokine-regulation in microglia are discussed below. In this review, we present relevant data showing that IL-6 induces dye uptake in DCs in a similar way to IFN-*γ* and is blocked by La^3+^, consistent with Cx HC-mediated response (Figure 4(b)). Interestingly, IL-6 antagonizes IFN-*γ*-induced dye uptake, which is correlated with its role in the maintenance of immature DCs [120]. This phenomenon might be promoted by downstream signaling pathways triggered by these cytokines that activate different suppressors of cytokine signaling proteins [121]. These data suggest that the effect of IL-6 on HC activity of DCs depends on the cytokine context present in the cellular microenvironment.

With these findings, it is plausible to anticipate that T-cell polarization is determined by the cytokine profile of the microenvironment, as well as by molecules directly exchanged and/or released to the extracellular milieu via GJCs and/or HCs, respectively, expressed by DCs and T cells.

### 2.2. Langerhans Cells (LCs)

These cells were described almost 150 years ago by Paul Langerhans [122], but their role remains elusive over almost 100 years until they were described as leukocyte derived cells [123]. LCs reside in skin epidermis and represent the first barrier against pathogens and external noxa [124]. Although LCs are less motile than dermal DCs [125], they are better APCs [126], suggesting their important role in antigen presentation. LCs are characterized by the expression of the nonpolymorphic class I MHC molecule CD1a and C-type lectin Langerin, as well as the presence of Birbeck granules, which are tennis-racquet-shaped intracytoplasmic organelles [127–129]. When LCs capture antigens, they migrate to skin draining lymph nodes (LNs) where they present antigens to naïve T cells [129] and might induce or suppress the immune response [130]. Early studies performed by Concha et al. observed at ultrastuctural level that physical interactions between LCs and T cells during allogeneic antigen presentation includes the presence of GJC-like structures [51, 52, 90].

Cx43 immunoreactivity was found in LC-like cells in human tissue with LC histiocytosis [91] and in MHCII^+^ epidermal LC-like cells from human epidermis [25]. However, Zimmerli et al. detect no Cx43 immunoreactivity in LCs (CD1a^+^ epidermal cells) from normal human skin [108]. This discrepancy could be explained in part by the inflammatory state of the tissue. Whether the tissue is under resting state or inflammation might affect the Cx43 expression, as occurs with the upregulation of Cx43 expression after stimulation in other immune cells. In support of the Cx expression, gap junctional communication between LCs has been shown to allow the transfer of antigenic peptides in a Cx43-dependent manner [25]. However, the possible functional expression of Cx HCs remains unknown.

Panx1 and Panx3 expression have been reported in murine epidermis [131], but their expression in LCs has not been documented. However, functional expression of Panx HCs is suggested by ATP-induced dye uptake in murine and human LCs [102, 132]. Since LCs express several purinergic receptors that contribute to the LC-mediated immune response [133], it is conceivable to suggest that Panx HCs might also contribute to cytokine release and activation of LCs.

### 2.3. Follicular Dendritic Cells

Unlike DCs, follicular DCs (FDCs) present a low phagocytic activity but high retention of antigen and immune complexes on their surfaces. They reside at follicles of secondary lymphoid organs [134], where they present antigens to B cells [135]. The origin of FDCs is a controversial topic because some evidences show that they emerge from bone marrow, while other studies propose that they derive from mesenchymal cells [134]. This controversy might have contributed to delay the establishment of primary cultures of FDCs and the subsequent demonstration of cell-cell communication mechanisms mediated by Cx- and Panx-based channels.

*In situ* hybridization studies showed Cx43 mRNA in human tonsils [93]. In addition, it was demonstrated that Cx43 colocalizes with FDC markers (CD21 and CD35) at germinal centers of human tonsils and spleen [91–93]. Moreover, gap junctional communication among FDCs and between FDCs and B cells has been demonstrated at functional and ultrastructural levels [91–93]. Here, we show that FDCs (CD11c^+^) found in mouse spleen follicles present Panx1 immunoreactivity (Figure 3). The expression of functional HCs on FDCs remains unknown, but currently it is possible to speculate that TNF-α [134], crucial cytokine for development of FDCs, might modulate the expression of GJCs and HCs, as it occurs in other APCs. Similarly, IL-6 might affect HC activity in FDCs because these cells are the main source of this cytokine at germinal centers [134].

### 2.4. Monocyte/Macrophages

Monocytes emerge from the same precursor of DCs in the bone marrow and circulate in the blood [98]. Upon tissue injury, they rapidly extravasate and differentiate in DCs or macrophages, depending on the cytokine pattern present in the microenvironment [136, 137]. Studies on GJCs in APCs started with demonstrations of gap junctional communication between macrophages [88, 89], and information on the expression of Cxs and Panxs in these cells is increasing progressively [55, 106, 138–143]. Recently, it was shown that tumor-associated macrophages express Cx43, and it seems that they form GJCs in long networks [139]. Similarly, alveolar macrophages form communicating networks with epithelial cells in the alveoli where they coordinate Ca^2+^ signaling [144]. This cell-cell communication might be protective effect because specific deletion of Cx43 in macrophages increases the release of proinflammatory cytokines [144]. In addition, monocytes and macrophages form heterocellular GJCs with CD103^+^ DCs, endothelial cells, and T cells [106, 140–142, 145].

Resting monocytes express Cx37 and, after activation, they also express Cx43. These Cxs regulate their adhesion and extravasation, respectively (Figure 2) [140, 142, 146, 147]. In support of this notion, TNF-α has been shown to increase Cx43 expression, adhesion, and extravasation of monocyte/macrophages [140, 142]. Treatment with TNF-α alone does not induce functional expression of GJCs in monocytes, but it remains to be demonstrated whether it induces Cx43 HC activity, which might be involved in cell adhesion [142, 146], as it has been demonstrated for Cx37 HCs [147]. IFN-*γ* does not induce the expression of HCs or GJCs but increases Cx43 levels, gap junctional communication, and* in vitro* migration when combined with LPS or TNF-α (Table 1) [140].

The expression of Panxs in monocytes was first suggested by ATP-induced dye uptake [148]. Recently, it was demonstrated that human monocytes express Panx1 under resting conditions, and its total levels are upregulated after treatment with LPS [138]. In monocytes, LPS induces functional expression of Panx1 HCs, which contributes to ATP release and consequently to IL-1*β* release [138].

Peritoneal, alveolar, and cell lines derived from macrophage express Cx37 and Cx43 under resting conditions, and upregulation of Cx43 expression is observed after activation [55, 72, 94, 144, 147, 149–155]. In macrophages, Cx37 negatively regulates cell adhesion as in monocytes [147], while Cx43 has been proposed to play a role in phagocytosis [150]. However, the latter remains controversial [153]. These particularities might rely on the different genetic background (mice strain, heterozygotes, or K.O.) and protocols used. Moreover, Cx43 HCs allow the release of small signaling molecules including ATP and NAD^+^ and also contribute to IL-1*β* release in macrophages infected with* Bacillus anthracis* [72, 149, 156]. In addition, it has been recently shown nitric oxide release through HCs [157] and thus, it is possible that Cx37 and/or Cx43 HCs allow nitric oxide release in activated monocyte/macrophages [158]. Macrophages also express Panx1 HCs, which are activated by extracellular ATP [159]. This finding was suggested previously in studies where HC blockers were shown to reduce the ATP-induced dye uptake in peritoneal macrophages and in a macrophage cell line [94, 152].

In macrophages, Panx1 HCs contribute to IL-1*β* release through a pathway independent of their permeability [37], but to our knowledge the possible functional regulation of these channels by cytokines has not been described. However, an interesting suggestion of the possible regulation of Panx1 by cytokines was investigated by gene expression pattern in macrophage polarization [160]. Macrophages present different phenotypes depending on the stimuli and the microenvironment cytokine signature. Then, “classic” activation of macrophages with LPS or cytokines such as TNF-α or IFN-*γ* leads to a proinflammatory profile, which is named M1 [161]. Conversely, “alternative” activation after exposure to IL-4, IL-10, or IL-13 or particular Toll-like receptor agonists leads to macrophage differentiation with an anti-inflammatory profile, which is named M2 [161]. Interestingly, while M1 polarization induces downregulation of Panx1 expression in macrophages, M2 polarization induces some upregulation [160]. These observations suggest the involvement of Panx1 in the anti-inflammatory response of M2 macrophages, but whether functional Panx1 HC activity is increased in M2 has not been published yet. Altogether, these data suggest that Cx and Panx HCs play an important role in macrophage activation; their possible regulation by pro- or anti-inflammatory cytokines is a vast unexplored field of research.

### 2.5. Kupffer Cells

Kupffer cells (KCs) are the largest population of resident macrophages in the body, the liver being their organ of residence [137, 162]. These cells have the ability to present antigens, undergo fusion, form large multinucleated cells, and induce Tregs activation and degradation of intravascular debris [137, 163–165]. Under resting state, KCs release anti-inflammatory cytokines promoting tolerance [163], but after stimulation they release proinflammatory cytokines and might present antigens to CD4^+^ (helper) and CD8^+^ (cytotoxic) T cells [166, 167].

**Figure 5:**
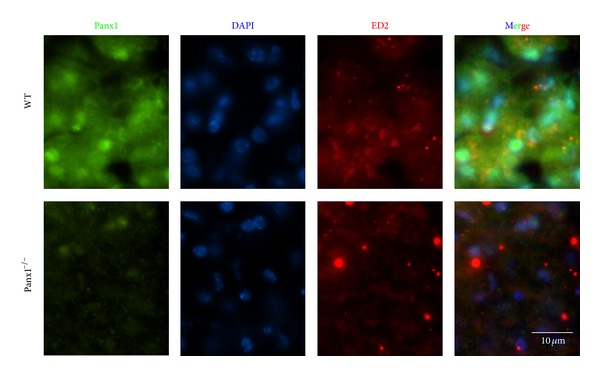
Expression of pannexin1 (Panx1) in Kupffer cells. Immunofluorescence analysis of liver cryosections (8 *μ*m thick) obtained from adult wild-type (WT) and Panx1^−/−^ adult (C57/BL6) mice was performed to analyze reactivity of Panx1 (green, primary antibody: rabbit anti-Panx1 antibody and secondary antibody goat anti-rabbit IgG conjugated to FITC) in ED2 (red: goat polyclonal antibody and secondary antibody mouse anti-goat conjugated to Cy3) positive cells, which correspond to Kupffer cells. Top panels correspond to a liver section of a WT mouse and bottom panels correspond to a liver section of a Panx1^−/−^ mouse. No specific Panx1 reactivity was detected in Panx1^−/−^ liver, but ED2 positive cells were evident. DAPI stain was used to visualize nuclei (blue), and merge is also shown. Panx1^−/−^ mice were kindly donated by Dr. Hanna Monyer (University of Heidelberg, Germany). Bar: 10 *μ*m.

In the liver, KCs are sparse, but under proinflammatory conditions they form aggregates and present increased Cx43 immunoreactivity at KC-KC interface, suggesting GJC formation* in vivo* [168, 169]. In support of this notion, cultured KCs express low Cx43 mRNA and protein levels but do not communicate through GJCs under resting conditions (Figure 2). However, after exposure to LPS/IFN-*γ*, cultured KCs enhance the expression of Cx43 (Table 1) that is located at KC-KC interface allowing gap junctional communication [168]. Neither Panxs nor Cx HCs have been demonstrated in KCs. Here, we show the presence of Panx1 in KCs recognized by their ED2 reactivity in wild-type mice (Figure 5). Consistently, Panx1 was not detected in ED2 reactive cells in liver sections of Panx1^−/−^ mice (Figure 5). Functional expression of Panx1 HCs in KCs and their possible regulation by cytokines still remain unknown.

### 2.6. Osteoclasts

Osteoclasts (OCs) are large multinucleated macrophages located in bones. They can be derived from bone marrow precursors or monocytes and have bone-resorbing activity [137, 162, 170]. Because autoimmune diseases lead to bone destruction (e.g., rheumatoid arthritis) [170], a rising interest in the study of the interplay between skeleton and the immune system (osteoimmunology) has taken place during the last decade. Several cytokines, including IL-17, type I and II IFNs, and receptor activator of nuclear factor kappaB ligand (RANKL), have the ability to induce osteoclastogenesis, the process that modulates bone remodeling [170, 171]. Conversely, under noninflammatory conditions, OCs present antigens to CD4 and CD8 T cells, which differentiate into regulatory T cells and inhibit bone resorption [171].

Cx43 mRNA and protein have been detected in cultured OCs and also at the bone (Figure 2) [172–182]. OCs derived from bone marrow precursors or monocytes that undergo fusion and form multinucleated tartrate-resistant acid phosphatase (TRAP) positive cells with bone-resorbing activity express Cx43, which contributes to fusion as observed by the use of Cx43 blockers [180, 181]. Considering the involvement of Cx43 in fusion of OC precursor and the fact that osteoclastogenesis is inhibited by osteoprotegerin released from stromal/osteoblast lineage cells [173, 175], it is possible that under normal conditions osteoprotegerin downregulates Cx43 and then prevents fusion of precursors. Interestingly, a cytokine member of the TNF family named RANKL induces osteoclastogenesis in combination with macrophage colony-stimulating factor (M-CSF) and also increases Cx43 expression (Table 1) [176].

Ultrastructural evidence of GJCs between OCs has been reported [177, 182], but the functional expression of GJCs has been only suggested. The contribution of GJCs to the bone-resorbing activity has been addressed by using HC blockers [178–181], but still leaving open the possibility that OCs may also express Cx or Panx HCs. For instance, the expression of Panx1 HCs could be feasible because these contribute to macrophage fusion, which leads to multinucleated cell formation [183]. In addition, immunofluorescence analysis of bones shows that most cells presented Panx3 at the growth plate [29], suggesting that OCs might express this protein. Finally, Cx43, forming either GJCs or HCs, is involved in the development of rheumatoid arthritis because silencing Cx43 in rat lower limbs reduces the number of OCs and delays the onset of this disease [174]. This suggests that Cx43 expression by OCs might contribute to the development of this disease and might be a relevant target for its treatment.

### 2.7. Microglia

Microglia, the main resident macrophage of the central nervous system, remove dead cells and monitor cell microenvironment. After injury or infection, activated microglia secrete proinflammatory cytokines and present antigens. In addition, deregulation of their activation is a hallmark of neurodegenerative diseases [184–186].

The study of Cxs and Panxs in microglia has been extensive. The expression of Cxs 32, 36, and 43 and Panx1 has been reported. Some of these proteins form functional GJCs and HCs that contribute to cell-cell communication, migration, and neuronal death (Figure 3) [41, 95, 96, 187–197]. In addition, the mRNA of Cx45 was found in mouse but was not detectable in human microglia [194]. Cx43 seems to play a relevant role because its total protein levels are upregulated in microglia activated by advanced glycation endproducts, amyloid-*β* peptide, DAMPs, PAMPs, cytokines, and a Ca^2+^ ionophore [96, 189, 192, 193, 195–197]. Indeed, microglia treated with advanced glycation endproducts, proinflammatory cytokines, PAMPs, and a Ca^2+^ ionophore form GJCs presumably constituted by Cx43 [192, 193, 195]. In support of this position, the specific blockade or lack of Cx43 in microglia of Cx43 K.O. mice abrogates the cytokine-induced GJCs [96, 196].

Gap junctional communication between microglia is tightly regulated by several cytokines (Table 1). In fact, intercellular communication mediated by GJCs is increased in microglia after treatment with TNF-α, TNF-α/IFN-*γ*, and TNF-α/IL-1*β* [96, 192, 196]. Shaikh et al. [192] demonstrated gap junctional communication after treatment with TNF-α in a microglial cell line, but a recent study performed by Sáez et al. [96] showed that TNF-α does not induce dye coupling in primary cultures of microglia. However, there are several differences that might explain this discrepancy: (1) one study evaluated dye coupling through scrape loading while the other used microinjection; (2) both studies used different TNF-α concentrations; and (3) one study used a microglial cell line and the other used primary cultures of microglia. Consequently, the interpretation of these results should be taken cautiously and the protocols reconsidered.

Recently, it was shown that extracellular ATP is required by the cytokine-induced GJCs and forces the early onset of this gap junctional communication [96], showing a synergistic effect between cytokines and DAMPs. As observed in DCs by Corvalán et al. [86], IL-6 prevents the induction of GJCs in microglia by preventing upregulation of Cx43 and Panx1, as well as by increasing free intracellular Ca^2+^ levels [96]. Furthermore, it is possible that IL-6 might disrupt cell adhesion between microglia as shown in other cells [198], and consequently it might also prevent the formation of GJCs. Recently, absence of dye transfer between microglia* in vivo* and between microglia and other brain cells has been shown in both resting and injury conditions [143]. This study assessed dye transfer by using sulforhodamine B and previous studies that demonstrated gap junctional communication in microglia used Lucifer yellow [96, 192, 193, 195, 196]. The difference in the method used to evaluate functional gap junctional communication is relevant because GJCs are selective to molecules with different size and charges. In particular, Cx43 GJCs are less permeable to cationic than anionic dyes [199–201]. In addition, microglial GJCs were recently identified at ultrastructural level* in situ* between microglia and neural cell progenitors and also with nonidentified cells [202]. These data correlate with immunoreactivity of Cx43 at sites of apposition between the aforementioned cells [202]. Finally, whether microglia establish GJCs* in vivo* allowing permeation of signaling or immunorelevant molecules remains controversial.

Recently, the expression of functional Cx and Panx HCs has been shown in microglia [73, 96, 187, 189, 203, 204]. Treatment with amyloid-*β* peptide increases Cx43 HC activity in microglial response, which in turn allows glutamate and ATP release [189]. Cx43 HC activity and ATP release are also increased by TNF-α/IFN-*γ*, but these two reactions are prevented by IL-6 [96]. These studies show that ATP is released through Cx43 HCs, although ATP might also be released by exocytosis [205]. In addition, Cx32 HC activity is increased in microglia treated with TNF-α and/or LPS, which induce glutamate release [187, 203, 204]. These findings suggest that a similar outcome in HC activity results from the action of different stimuli that trigger different intracellular signaling cascades.

A similar mechanism commands Panx1 HC activity, which can be enhanced by amyloid-*β* peptide and contributes to glutamate and ATP release [189]. In addition, TNF-α/IFN-*γ* increases Panx1 HC activity, leading to ATP release [96]. Moreover, microglial Panx1 HCs present an increased activity after exposure to high concentrations of ATP, which favor microglial migration [96, 190, 191]. Although exposure to TNF-α/IFN-*γ* or TNF-α/IL-1*β* does not affect the basal ATP-induced HC activity in microglia, IL-6 prevents the induction of Panx HC activity in cells treated with proinflammatory cytokines [96]. This inhibitory effect of IL-6 might downregulate microglial migration, as shown by arachidonic acid that closes Panx1 HCs [188]. Conversely to migration, Panx1 does not contribute to microglia proliferation at embryonic stages [206]. To sum up, these results suggest that microglia might migrate toward amyloid-*β* peptide plaques or ATP foci in a Panx1-dependent manner.

In addition, several studies show increased dye uptake or molecule release (e.g., ATP, glutamate) in activated microglia [192, 204, 207–209], but the use of Cx and Panx HC blockers (e.g., carbenoxolone) does not dissect the molecular entity that mediates the dye uptake. However, these experiments unveil that Cx and Panx HCs may contribute to neuronal death and host defense against pathogen infections. The latter seems to be mediated by IL-1 [208, 209]. In addition, recent studies show that HC blockers delay the development of Alzheimer' disease, amyotrophic lateral sclerosis, and multiple sclerosis in murine models of these diseases [204, 207], suggesting that HC blockers might be useful as a therapeutical approach to the treatment of these diseases. Interestingly, it was shown that carbenoxolone delays the onset of multiple sclerosis in mice by preventing the release of IL-23 from microglia and the polarization of Th17 cells [210]. Related to this last study, it may be possible that microglia communicate with T cells through Cx- and Panx1-based channels, determining the polarization of T cells. However, the heterocellular expression of GJCs between microglia and T cells, or the regulation of Cx- and Panx-based channels by IL-17, has not been addressed yet.

### 2.8. Neutrophils

These circulating leukocytes are the most abundant in the blood (50–70%), the first cells that arrive at the injury site after detection of chemokines and cytokines, and the first responders to most injuries sites. In addition, a new role has been shown in the maintenance of long-lived B cells by interacting at marginal zone in spleen [211, 212]. Although neutrophils express low or no MHC II and costimulatory molecules under resting conditions exposure to different cytokines, as occurring in chronic pathologies, leads to upregulation of MHC II expression in neutrophils and they acquire APC characteristics [213, 214]. Moreover, neutrophils perform MHC I-mediated cross-presentation and MHC II-mediated antigen presentation to T cells [214, 215]. In addition, murine neutrophils act as APCs and contribute to Th1 and Th17 cells polarization* in vitro *in absence of exogenous cytokines, and as expected those effects were MHC II-dependent [216]. Importantly, neutrophil-T-cell interaction promotes Th17 cell polarization independent of TGF-*β* and IL-6, suggesting that contact-dependent intercellular communication plays an important role in this process [216]. Thus, it is currently considered that neutrophils participate not only in early stages of innate immune responses, but also in further stages of adaptive immune responses, making their cellular interactions key steps for coordinating immune responses.

The study of Cxs in neutrophils began just two decades ago, and now it has been expanded to Panxs. Although no Cxs are detected in mouse or human circulating neutrophils, they expressed Cxs 37, 40, and Cx43 at mRNA and protein level after activation [17, 217–219]. However, some studies did not detect Cx43 in human blood neutrophils [220, 221]. However, this was expected considering that neutrophils were not stimulated.

Neutrophils form aggregates and communicate to each other through GJCs only after LPS or TNF-α exposure and in the presence of a cytokine containing endothelial cell-conditioned medium [217]. Nevertheless, the exact cytokine (or cytokine mixture) that induces expression of GJCs in neutrophils remains unknown. Additionally, neutrophils form functional GJCs with endothelial cells, which favor their neutrophil migration [142, 219]. In fact, there is ultrastructural evidence of gap junction formation between neutrophils and endothelial cells after ischemic injury [141]. Interestingly, TNF-α increases the neutrophil adhesion to endothelial cells as well as the migration* in vivo* in a Cx43-dependent manner [142]. However,* in vitro* studies have shown that TNF-α reduces the gap junctional communication between these cells [219], probably through a downregulation of endothelial Cxs. However, this apparent controversy might be due to differences between* in vivo* and* in vitro* studies, as well as the endothelial cell type used, timing of the response, stage of recruited neutrophil, and differences in microenvironment signals that command the inflammatory process. Similar differences occur in studies of neutrophil interactions with epithelial cells. While* in vivo* studies show that Cx43 contributes to neutrophil migration across an alveolar epithelial barrier in response to LPS [220],* in vitro* studies show absence of gap junctional communication between neutrophils and airway epithelial cells [221]. In addition, and supporting the contribution of Cx43 to cell-cell communication between the endothelium and neutrophils during extravasation, in several studies downregulation of Cx43 reduces levels of neutrophil extravasation after burn injury, wound healing, and spinal cord damage [222–224]. Conversely, Cx40 deletion did not affect neutrophil migration [225], and the contribution of this protein to neutrophil activation is still unknown.

The expression of Cx and Panx HCs has been demonstrated in neutrophils. After activation, neutrophils present Cx43 reactive puncta on their surface [217] and release ATP through Cx43 HCs that favor migration without effect on adhesion to endothelial cells [218, 219]. Moreover, Panx1 HCs play a key role during neutrophil chemotaxis because their surface expression is polarized toward the leading edge where they allow ATP release and thus provide guidance for neutrophil migration [34, 226, 227]. It remains to be studied whether cytokines regulate Cx or Panx HC activity in neutrophils.

### 2.9. B Cells

B cells are also APCs because they present antigens in MHC II to CD4^+^ T cells, which induce antibody production [135]. During this activation, B cells polarize toward the synapse, which determines whether the cell becomes effector or memory B cell [228, 229].

Expression of Cxs 40 and 43 has been demonstrated in isolated human B cells and at germinal centers of tonsil [92, 93]. Cx43 is also expressed in splenic B cells and some cell lines [230, 231]. Although endogenous functional expression of HCs remains unknown, Cx43 overexpression increases membrane permeability in a B cell line as expected [232]. Cx43 contributes to B cell spreading and adhesion. In fact, mutations that block the channel function of Cx43 impair the B-cell receptor- (BCR-) mediated spreading [230, 232]. However, Cx43 mutant expressed by B cells retained the ability to rearrange the cytoskeleton, conversely to B cells expressing a Cx43 with deletion of C-terminal. Unexpectedly, in this study no increase in dye uptake in resting or activated wild type B cells was found. In addition, blockade of HCs did not produce changes in BCR-induced cell spreading [232], suggesting that in these cells Cx43 contributes with a role to the intracellular signaling. It is worth mentioning that Cx43 colocalizes with actin in B cells and acts as a downstream signal for CXCL12-induced activation of Rap1 [233]. Moreover, downregulation of Cx43 impairs the CXCL12-induced migration and transendothelial migration [233], but whether HC activity contributes to B cell migration has not been studied yet.

**Figure 6:**
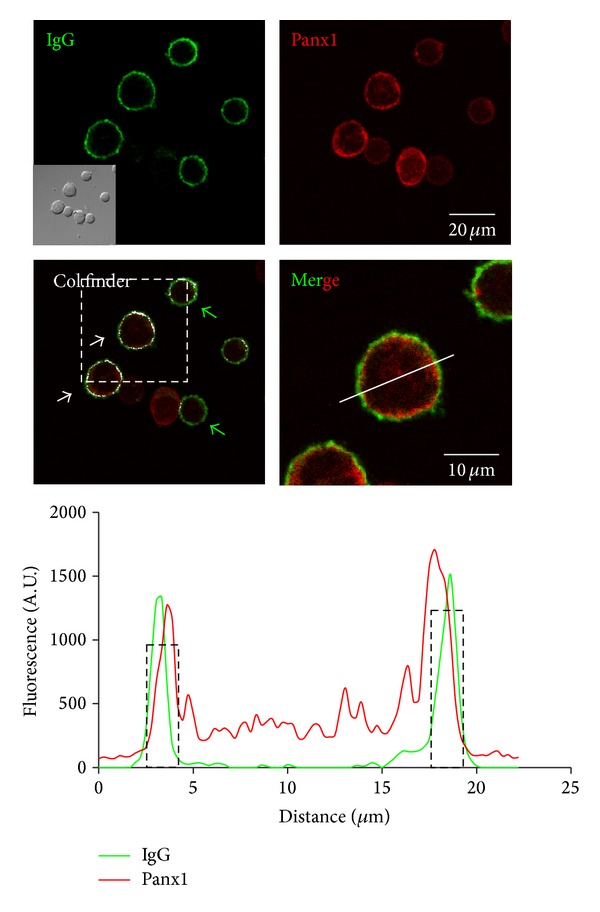
B cells present pannexin1 at the cell surface. Confocal images (Olympus, FluoView FV1000) of immunofluorescence analysis of freshly isolated B cells fixed in ethanol (70%). B cells were isolated from peripheral lymph nodes by positive selection from balb/c mice. Top left: B cells were identified with IgG (conjugated to FITC, green); the inset shows the bright field. Top right: pannexin1 (Panx1) immunoreactivity (red, primary antibody: rabbit anti-Panx1 antibody and secondary antibody goat anti-rabbit IgG conjugated to Cy3) is shown. Bar: 20 *μ*m. Middle left: using ImageJ colocalization finder, it can be seen that Panx1 colocalizes with IgG (white) at the cell surface in some B cells (white arrows). B cells with low or no colocalization are indicated (green arrows). Middle right: zoom and merge of IgG and Panx1 labeling in a B cell denoted by a dotted square at middle left panel. The white line denotes the region used for the line scan. Bar: 10 *μ*m. Bottom: ImageJ line scan analysis shows the fluorescence intensity of each channel through the white line in the middle of each cell. The peak coincidence (denoted by dotted squares) is an index of colocalization between the different fluorophores.

Panx1 expression in B cells has not been reported, and unlike T cells there is no further evidence to suggest that B cells increase membrane permeability under certain conditions. Here, we present evidence that Panx1 is expressed in B220^+^ B cells in mouse spleen (Figure 3). Moreover, freshly isolated murine B cells present Panx1 at the cell surface, suggesting that it might form functional HCs (Figure 6). Finally, although ATP stimulation did not induce dye uptake in B cells, it remains to be demonstrated whether antigen triggering affects the activity of Panx1 HCs.

Early studies showed the formation of GJCs between B cells and T cells that contribute to IgM synthesis [231], and also between B cells and FDCs [92, 93], suggesting a role for GJCs in B cell activation at immunological synapses. However, it is still unknown whether cytokines affect GJCs or HCs in B cells. Moreover, it remains to be elucidated whether other soluble cytokines such as IL-6, APRIL, BAFF, and TNF-α regulate the functional state of GJCs and/or HCs.

## 3. Concluding Remarks

**Figure 7:**
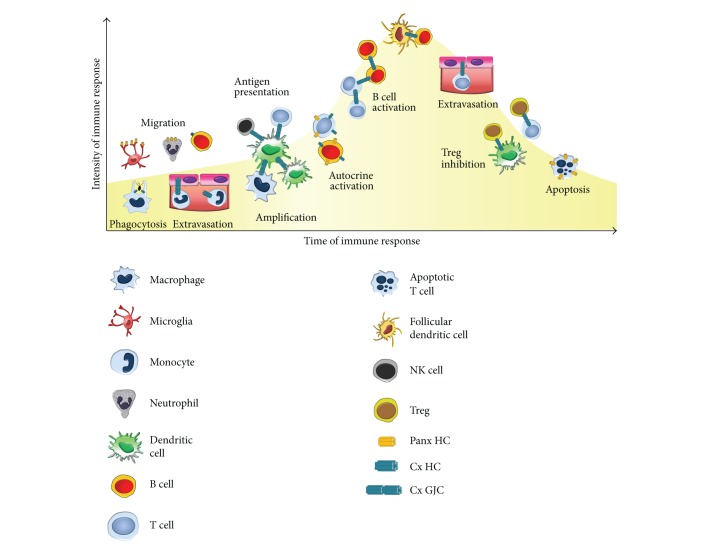
Scheme of different stages of classical immune response. The reported role for connexin- and pannexin-based channels is depicted in different immune cells functions as migration, antigen presentation, clonal expansion, and apoptosis.

The immune response efficiency relies on several homocellular and heterocellular interactions, which provide amplification to this response. Immune cells use different types of cellular communications, such as cytokines [3], exosomes [234], tunneling nanotubes [235], GJCs [17], and HCs. As shown here, all APCs express Cxs and/or Panxs and, in general, they are upregulated or redistributed after activation. GJCs and HCs contribute to almost all stages of the classical innate and adaptive immune response (Figure 7).

After injury, GJCs and HCs contribute to leukocyte extravasation [140–142, 146, 147, 219]. Panx1 HCs contribute to the recruitment of neutrophils and microglia toward the injury site [191, 227]. Although it remains controversial, it has been proposed that Cx43 contributes to phagocytosis [158]. Moreover, activated DCs, monocytes, macrophages, neutrophils, and microglia can communicate through GJCs [24, 86, 87, 96, 168, 176, 192, 196, 217], and HCs have been demonstrated in some of them. At this step, gap junctional communication might amplify the immune response because APCs might share specific information as antigen peptides [24, 25], which will increase the number of responding cells. Migratory DCs that arrive to lymph nodes present increased levels of Cx43 and Cx45 [86]. Recently, the expression of functional GJCs between DCs and T cells during immune synapse was shown to contribute to T-cell activation [13, 61], as it was previously suggested (Figure 7) [42, 52, 90, 236]. Prior to DC-T cell interaction, guidance of T-cell migration by extracellular signals induce specific Ca^2+^ dynamics that allow the establishment of kinapses and synapses, which correspond to short and long lasting interactions between these cells, respectively [237, 238]. Interestingly, recently it was shown that paracrine purinergic signaling modulates Ca^2+^ signaling in T cells in a P2X_4_ and P2X_7_ receptor-dependent manner, which ultimately reduce their motility [239]. Then, it is possible to anticipate that HCs might contribute to ATP release from mature DCs, which in the lymph nodes will help to establish DC-T-cell contact leading to antigen presentation.

It has been reported* in vitro* as well as* in situ* that human naïve CD8^+^ T cells establish GJCs with melanoma target cells, contributing to their activation, but not to their lytic function [240]. Conversely, human NK cells establish GJCs with DCs and tumor cells in a Cx43-dependent process that contributes to NK cell-mediated lysis and further antitumoral immunity (Figure 7) [107]. Moreover, GJCs between polarized T cells (Th1 or Th2) have also been demonstrated [145]. Interestingly, Th1 and Th2 cells form GJCs with macrophages, but Th2 cells present lower levels of Cx43 [145], suggesting the possible involvement of other Cxs in this process. Similar to Th2 cells, in Th17 cells Cx43 is absent [241]. However, the expression of GJCs in these cells has not been shown. In addition, here we showed that two polarizing cytokines (IFN-*γ* and IL-6) induce HC activity, but in combination they have antagonistic effects. This last fact is very important because it suggests that Cx GJCs and HCs might be involved in Th polarization, and different Cx profiles could be associated with a different phenotype.

During T-cell activation, expression of GJCs and HCs mainly constituted by Cx43 contributes to T-cell proliferation [81, 82, 231, 242]. In addition, it has been recently demonstrated that T cells also express functional Panx1 HCs during activation [243–246]. Indeed, GJCs are formed during T cell-B cell interactions [231, 247], as well as between B cells [231, 247], promoting immunoglobulin secretion. Here, we show that Panx1 is at the cell surface of B cell and might form HCs that might contribute to B cell activation. To produce high affinity antibodies, B cells must interact with FDCs, and GJCs contribute to this process (Figure 7) [92, 93].

In the peak of an immune response, lymphocytes should arrive at the affected tissue where GJCs are observed between T cells and endothelial cells (Figure 7) [248]. Also, Cx43 contributes to B cell spreading and adhesion [230, 232]. Consequently, it is possible that Cx43 and GJCs might be involved in this process* in vivo*. Moreover, Cx43 contributes to the development of Tregs [241], which transfer cAMP through GJCs and inhibit T-cell activation during resolution of immune response or immune suppression by Tregs [12]. Interestingly, GJCs between Tregs and DCs prevent the development of contact hypersensitivity reactions mediated by CD8 T cells [15]. Modulation of immune responses using “educated” immune cells was recently used to prevent allergy reactions in mice. This effect was based on the generation of tolerogenic DCs after gap junctional communication with Tregs [249]. Another recent study showed that Tregs through GJCs are involved in controlling the HIV replication in T cells [250], opening an unexplored way to modulate the HIV infection.

We have summarized data showing that cytokines regulate both GJCs and HCs, which participate during most, if not all, steps of adaptive immune response. GJCs seem to be involved mainly in antigen presentation, whereas HCs are involved in functions such as migration or autocrine and paracrine activation. As presented here, during the lasts years a rising interest by immunologist in the field of cell-cell communication mediated by Cx- and Panx-based channels has driven many of the developments in the field. However, there is still much work to do because of more required technology transfer and collaboration between immunologists and “gap junctionologists.” When the latter occurs, the GJC and HC regulation by cytokines might be used to provide an efficient immune response or to prevent or inhibit deleterious immune activation. Until recently, an important issue was the lack of specific tools to evaluate the role of GJC and HC activity* in vivo* during the immune response. A first interesting approach was used with the reconstitution of a mice previously irradiated [251]. In this chimeric mouse, a slight effect was observed during inflammation, and no gene dosage was observed [251], suggesting the possibility of gene compensation. However, recently two different murine models were developed to study the role of Cx43 in CD11c^+^ cells, such as DCs and macrophages [106, 144]. These studies used* in vivo* imaging and tissue analysis to show the relevance of gap junctional communication between APCs and APCs or between APCs and epithelial cells [106, 144]. These tools have started a new age in the study of Cx43 in the immune response, even when a cell-specific K.O. for Panxs is still missing. However, compensation by other proteins might occur in these mice because the immune response should not rely only on the function of one protein, so the use of these tools should be analyzed in depth to avoid misinterpretations.

Finally, there is another possibility for the use of specific drug delivery to inhibit GJCs and HCs during* in vivo* responses, but the field of Cx- and Panx-based channel blockers is under development and mimetic peptides are not much specific [252]. However, new approaches are rising, such as with the antibody Cx43^(E2)^ which inhibits Cx43 HCs [253, 254]. After the development of these and other tools, the regulation by cytokines will open new possibilities to adjust the innate and adaptive immune responses.

## Figures and Tables

**Table 1:** Effect of different cytokines on GJCs and HCs in different antigen-presenting cells.

Cytokine(s)	Cell type	Effect on Cx or Panx expression	Effect on GJCs, HCs and technique used
TNF-α	DCs	=Cx43 (Wb) [24]	=GJCs (DT) [24, 86]
		+MCL: ↑Cx43 (Wb) [24]	+MCL ↑GJCs [24]
	Mo	NE	=GJCs (DT) [140]
	Microglia	↑Cx32 (FC) [187]	↑HCs (MR, DU) [187, 192]
		↑Cx43 (Wb) [192]	↑GJCs (SL) [192]
		+ATP: ↑Cx43, Panx1 (Wb) [96]	+LPS ↑GJCs [196]
			=GJCs [96] +ATP ↑GJCs (DT) [96] +ATP ↔HCs (DU) [96]
	Neutrophils	+CM: ↑Cx37, 40, 43 (Wb, IF) [217]	+CM ↑GJCs (DT) [217]

IL-1*β*	DCs	NE	=GJCs (DT) [86]
	Microglia	NE	=GJCs (DT) [96]

IFN-*γ*	DCs	=Cx43 (Wb) [87]	=GJCs (DT) [86]
			↑HCs (DU)
			+LPS ↑GJCs [87]
	Mo	NE	=GJCs (DT) [140]
			+LPS ↑GJCs [140]
	KCs	=Cx43 (RT, Wb) [168]	+LPS ↑GJCs (DT, IF) [168]
	Microglia	NE	=GJCs (DT) [96]

IL-6	DCs	NE	=GJCs (DT) [86]
			↑HCs (DU)
	Microglia	NE	=GJCs (DT) [96]

RANKL	OCs	NE	↑GJCs? ↑HCs? [178]

CXCL12	B cell	↑Cx43 Phosphorylation (Wb)	=HCs

RANKL/M-CSF	OCs	↑Cx43 (RT, Wb) [176]	↑GJCs (IF) [176]

IFN-*γ*/IL-6	DCs	ND	=HCs (DU)

TNF-α/IL-*β*	DCs	↑Cx43 (RT, Wb) [86]	↑GJCs (DT) [86]
	Microglia	↑Cx43, Panx1 (Wb) [96]	↑GJCs (DT) [96]

TNF-α/IFN-*γ*	DCs	ND	=GJCs (DT) [86]
	Mo	↑Cx43 (Wb) [140]	↑GJCs (DT, IF) [140]
	Microglia	↑Cx43, Panx1 (Wb) [96]	↑GJCs (DT) [96, 196] ↑HCs (DU) [96]

TNF-*α*/IL-*β*/IFN-*γ*	DCs	↑Cx43 (RT, Wb) [86]	↑GJCs (DT) [86]

TNF-*α*/IL-*β*/IL-6	DCs	NE	↓GJCs (DT) [86]
	Microglia	↓Cx43, Panx1 (Wb) [96]	↓GJCs (DT) [96] ↓HCs (DU) [96]

TNF-*α*/IFN-*γ*/IL-6	DCs	NE	↓GJCs (DT)
	Microglia	↓Cx43, Panx1 (Wb) [96]	↓GJCs (DT) [96] ↓HCs (DU) [96]

TNF-*α*/IL-1*β*/IFN-*γ*/IL-6	DCs	NE	↓GJCs (DT) [86]

CM: conditioned medium, DCs: dendritic cells, DT: dye transfer, DU: dye uptake, FC: flow cytometry, IF: immunofluorescence, KCs: Kupffer cells, LPS: bacterial lipopolysaccharide, MCL: melanoma cell lysate, Mo: monocyte, NE: not evaluated, OCs: osteoclasts, RT: reverse transcription polymerase chain reaction, SL: scrape loading, and Wb: Western blot. Effect on HC or GJC activity: no effect (=), upregulation (↑), and downregulation of induced activity (↓).
